# Gel-free sample preparation techniques and bioinformatic enrichment analysis to in depth characterise the cell wall proteome of mycobacteria

**DOI:** 10.1016/j.mex.2018.05.013

**Published:** 2018-05-25

**Authors:** Clemens Hermann, Alexander D. Giddey, Andrew J.M. Nel, Nelson C. Soares, Jonathan M. Blackburn

**Affiliations:** Department of Integrative Biomedical Sciences, Institute of Infectious Disease & Molecular Medicine, Faculty of Health Sciences, University of Cape Town, Anzio Road Observatory, Cape Town 7925, South Africa

**Keywords:** Cell wall enrichment and bioinformatic enrichment analysis, Cell wall proteomics, Mycobacteria, Proteomics, Bioinformatic enrichment analysis

## Abstract

The comprehensive characterisation of the cell wall proteome of mycobacteria is of considerable relevance to both the discovery of new drug targets as well as to the design of new vaccines against *Mycobacterium tuberculosis*. However, due to its extremely hydrophobic nature, the coverage of proteomic studies of this subcellular compartment is still far from complete. Here, we report novel gel-free cell wall sample preparation procedures and quantitative LC–MS/MS measurements on a Q Exactive mass spectrometer. We combine these with a novel post-measurement bioinformatic analysis to filter out likely cytosolic contaminants. This reveals a subset of proteins that are highly enriched for cell wall proteins. The success of this approach is verified by peptide-centric measurement of the abundance of known subcellular markers, as well as analysis of the percentage of predicted membrane proteins within the purified fraction. While *M. smegmatis* was used during this study to establish and optimise the sample preparation procedures, these can easily be applied to other mycobacterial species, such as *M. bovis* BCG or *M. tuberculosis*.

• Improved gel-free cell wall sample preparation gives higher yields of tryptic peptides for LC–MS/MS measurement.
• Higher yields of tryptic peptides provide better quantitation and coverage of cell wall proteome.
• Post-measurement enrichment analysis filters out high abundance cytosolic contaminants that have carried through the experimental analysis.

## Graphical abstract

**Figure:**
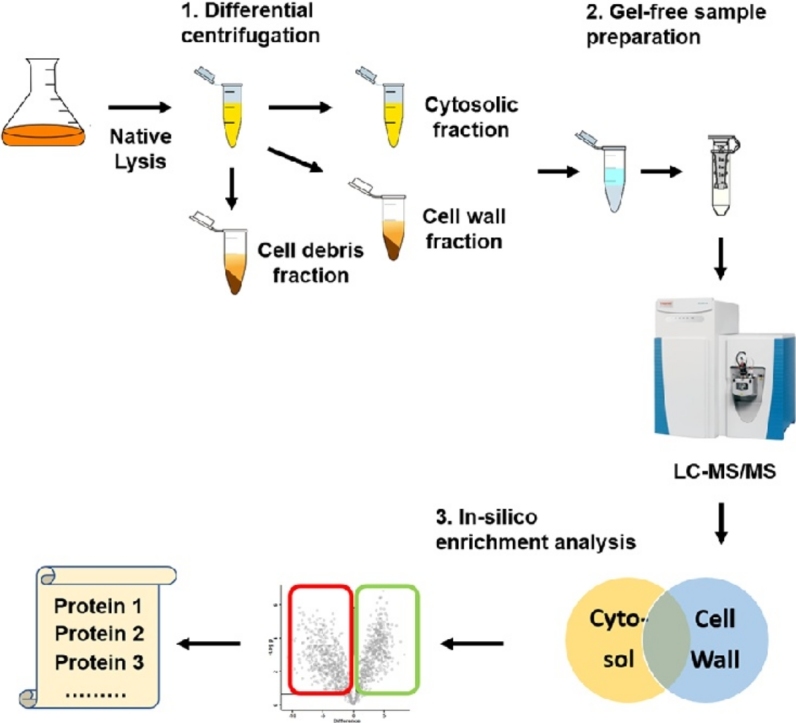

**Specifications Table**

**Table:** 

Subject area	Immunology and Microbiology
More specific subject area	Cell wall proteomics
Method name	Cell wall enrichment and bioinformatic enrichment analysis
Name and reference of original method	Partly based on previous methods [[1], [2], [3]] This new cell wall proteomics approach was applied to a biological question in our recent study [4]
Resource availability	The dataset used in this publication is part of a larger dataset freely accessibly on PRIDE (Accession Number: PXD008075). http://www.biochem.mpg.de/5111795/maxquant http://www.biochem.mpg.de/5111810/perseus

## Method details

### Introductory remarks

The study of the cell wall proteome of mycobacteria is an active field of research, given its attractiveness for drug and vaccine targets, with a number of studies and protocols being published for *M. tuberculosis* laboratory strains H37Rv [[5], [6], [7]] and H37Ra [8], clinical strains of *M. tuberculosis* [9], *M. bovis* BCG [3,5], *M. marinum* [10] and *M. smegmatis* [11,12]. While these mycobacterial species differ in their pathogenicity and environmental habitat, which may uniquely shape the composition of the cell wall proteome, this protocol – although optimised using the fast-growing mycobacterial species *M. smegmatis* as a surrogate – can be directly applied to all mycobacterial species.

**Fig. 1:**
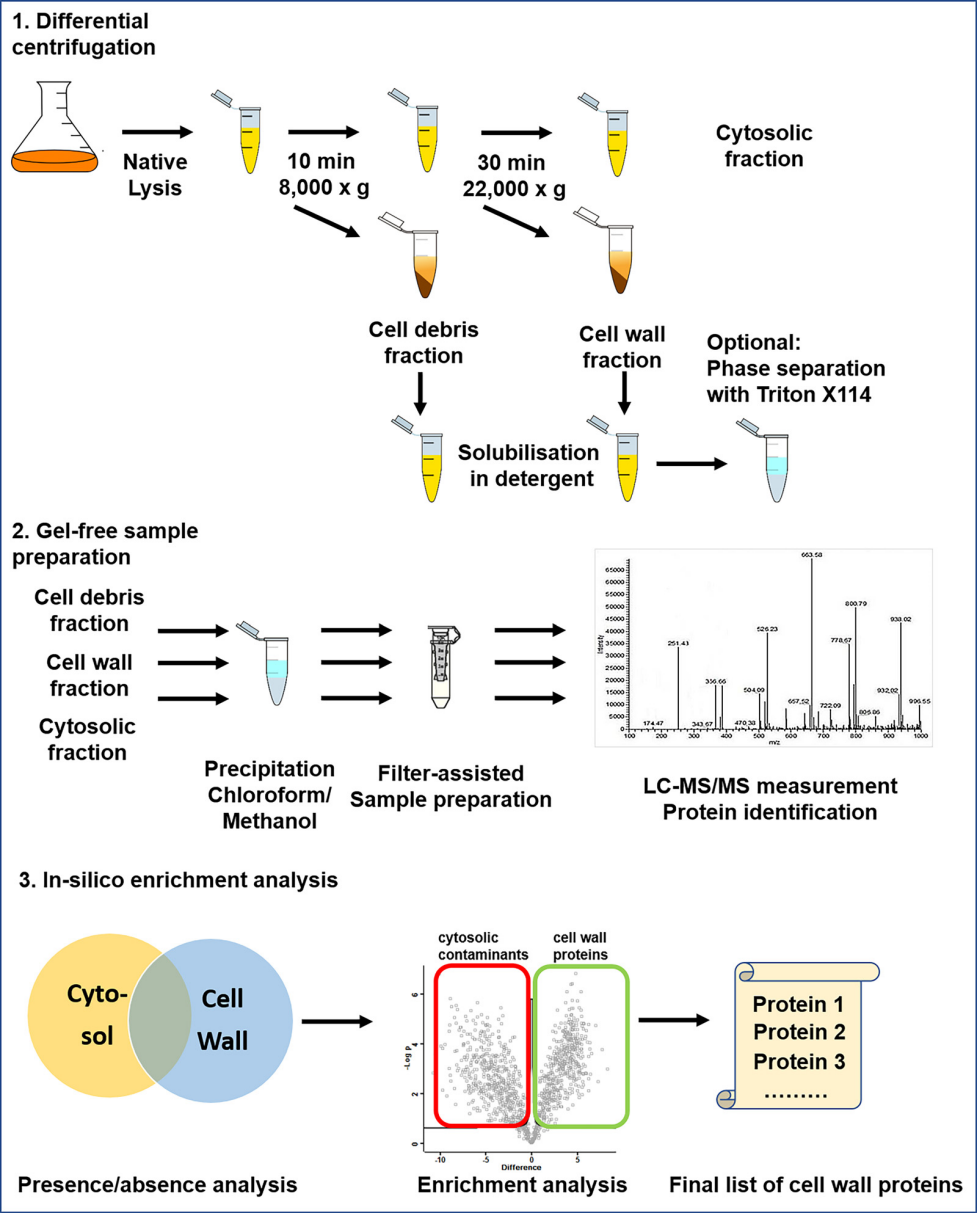
Overall workflow of cell wall proteomics approach.

A major hurdle to obtaining complete coverage of the cell wall proteome of mycobacteria is the high hydrophobicity of the cell wall itself. This poses a major challenge for the complete extraction of cell wall proteins from the cell wall, as well as for the efficient generation of tryptic peptides from hydrophobic proteins. Historically, different experimental approaches have been used to characterise the cell wall proteome of mycobacteria. These include differential centrifugation [1,2], phase separation using Triton X114 [1,3], cell surface protein biotinylation followed by enrichment with magnetic streptavidin beads [13], trypsin shaving [11] as well as detergent extraction of outer membrane proteins [10]. Most of these approaches rely on gel-based separation prior to tryptic digestion and analysis by mass spectrometry; all of them suffer from the presence of high-abundance cytosolic contaminants in the cell wall fraction. To overcome this challenge, we have developed sample preparation procedures and a novel downstream data analysis method that together enable the comprehensive identification and quantitation of the cell wall proteome of mycobacteria. Our method comprises three steps: differential centrifugation, gel-free sample preparation and *in silico* enrichment analysis (Fig. 1). We have successfully applied this method to quantify the changes in the cell wall proteome of *M. smegmatis* after exposure to rifampicin [4].

### Growth and harvest of M. smegmatis

Only MilliQ water should be used for any buffers and aqueous solutions during the protocol. Likewise, all plastic tubes should be mass spectrometry compatible. Growth condition will vary according to the experimental question being asked for each individual study. We used standard growth condition for optimisation of the current procedure. The *M. smegmatis* strain mc^2^ 155 was grown in 7H9 Middlebrook broth (BD, Maryland, USA) supplemented with 0.1% Tween-20, 0.2% glycerol (v/v) and 10% OADC (Becton Dickinson) at 37 °C with constant agitation at 120 rpm. Cells were grown until mid-log phase (OD_600_ ∼1.2), harvested by centrifugation at 3,500 × *g* for 10 min at 4 °C and cell pellets were subsequently washed three times with phosphate-buffered saline pH 7.4 (PBS).

### Cell lysis and enrichment of cell wall fractions

Cell pellets were lysed in native lysis buffer (PBS pH 7.4), supplemented with complete protease inhibitors (Roche, Germany), by six cycles of sonication for 30 s with cooling on ice between cycles. Alternatively, bead beating could be used in place of sonication for lysis. The total cell lysate was subjected to centrifugation at 8000 × *g* for 10 min to pellet cell debris (CD) and the clarified lysate was filtered through a 0.2 μm filter in accordance with institutional health and safety guidelines. The pelleted cell debris were washed once with PBS and resuspended in 0.1 M Tris, 2% SDS and 100 mM DTT, pH 8.5, and boiled at 95 °C for 30 min. The cell debris samples were cooled to room temperature on ice, resuspended in a final concentration of 6 M Urea with shaking for 30 min at room temperature and kept for further analysis. The cell wall fraction (CW) was pelleted from the lysate by centrifugation at 22,000 × *g* for 30 min at 4 °C. Depending on equipment available, this centrifugation step can be increased to 27,000 × *g* for 30 min to increase coverage of the cell wall fraction or 100,000 × *g* for 1–2 h to pellet the plasma membrane as well. The supernatant representing the cytosolic fraction was kept for further analysis. The cell wall pellet was washed twice with PBS (pH 7.4) and resolubilised in a detergent of choice shaking for 2 h at 4 °C. We have successfully used glycosides such as 1% dodecyl-D-maltoside, non-ionic and anionic detergent mixtures such as 0.1% Triton X100, 0.05% Tween-20 and 0.2% CHAPS or 0.15% deoxycholate and 0.1% SDS, as well as 2% Triton X114. We have shown that each detergent favours the extraction of specific protein classes – glycosides such as DDM are good solubilisers of porins, whereas the Triton family detergents are good at solubilising more hydrophobic proteins such as lipoproteins [4]. Therefore, the choice of detergent will be dependent on the experimental question. After resolubilisation, insoluble aggregates were removed by centrifugation at 22,000 × *g* for 10 min. If Triton X114 was used for solubilisation, a phase separation step can be performed by incubation of the samples at 37 °C for 15 min [3]. If this is desired, the aqueous phase is discarded and the detergent phase is backwashed twice with PBS. Protein concentration of each fraction (cell debris fraction, cell wall fraction, cytosolic fraction) was determined by RCDC assay (Bio-Rad). To remove any detergents that may interfere with subsequent analysis by LC–MS/MS, total protein from each subcellular fraction was precipitated by addition of methanol-chloroform as described previously [14]. It must be emphasised that this step should not be performed in plastic reaction tubes, but in glass vial of the appropriate size to avoid contamination with polyethylene glycol or other plasticisers which will interfere with subsequent chromatography as well as mass spectrometry measurements. The air-dried protein pellet was solubilised in 8 M urea, 0.1 M Tris (pH 8.5).

### Gel-free sample preparation using filter-aided sample preparation

One major hurdle of cell wall proteomics for mycobacteria is sample loss as a consequence of using gel-based sample preparation techniques that are typically required for sufficient detergent removal. We employed gel-free sample preparation procedures that have been developed previously for removal of detergents [15]. For this purpose, a minimum of 50 μg of each cell fraction was transferred into a Ultracel 30,000 MWCO centrifugal unit (Amicon Ultra, Merck) and concentrated by centrifugation at 14,000 × *g* for 15 min. The samples were washed three times with buffer UA (8 M urea, 0.1 M Tris, pH 8.5), reduced with 100 mM DTT for 30 min at room temperature and excess DTT was removed by centrifugation for 15 min at 14,000 × *g*. Iodoacetamide (0.05 M, final concentration) was added to each sample and incubated in the dark at room temperature for 20 min. Excess iodoacetamide was removed by three rounds of buffer exchange with UA buffer followed by three rounds of buffer exchange into ABC buffer (0.05 M ammonium bicarbonate pH 8.0, 20 mM CaCl_2_). Depending on the trypsin used, the addition of 20 mM CaCl_2_ can optionally be omitted. Sequence-grade Trypsin (NEB) was added to the samples at an enzyme to protein ration of 1:50 – 1:100 and incubated in a wet chamber overnight (at least 18 h). Tryptic peptides were eluted by three rounds of buffer exchange with ABC buffer and desalted using home-made STAGE tips that contain Empore Octadecyl C18 solid-phase extraction disks (Supelco). C18 disks were activated with three washes with solvent B (80% acetonitrile, 0.1% formic acid) and equilibrated with three washes with solvent A (2% acetonitrile, 0.1% formic acid). Tryptic peptides (10 μg) were loaded on the C18 disc and the disc then centrifuged at 4000 × *g* for 1 min before being washed three times with solvent A. Peptides were then eluted three times with solvent C (60% acetonitrile, 0.1% formic acid) into glass capillary tubes. Eluted peptides were dried under vacuum and resuspended with solvent A to a concentration of 200 ng/μl.

### LC–MS/MS analysis

We used standard procedures for LC–MS/MS analysis of the tryptic peptides. Briefly, separation of peptides by liquid chromatography was performed on an Ultimate 3500 RSnano UPLC system (Dionex) using a home- made precolumn (100 μm ID × 20 mm) connected to an analytical column (75 μm × 200 mm) packed with C18 Luna beads (5 μm diameter, 100 Å pore size; Phenomenex 04A-5452). Desalted peptides (200 ng per sample) were loaded onto the column with a starting mobile phase of 2% ACN, 0.1% formic acid and separated at a constant flow rate at 300 nL/min by the following optimised gradient: 10 min at 2% ACN, increase to 6% ACN for 2 min, to 60% ACN over 60 min, to 80% ACN over 5 min, followed by a column wash of 80% for 13 min. Mass spectra were collected on a Q Exactive mass spectrometer (Thermo) in a data-dependent manner with automatically switching between MS and MS/MS scans using a top-10 method. Peptides were ionised by electrospray ionisation and MS spectra were acquired at a resolution of 70,000 with a target value of 3 × 10^6^ ions or a maximum integration time of 250 ms. The scan range was restricted between 300 and 1750 *m/z*. Peptide fragmentation was performed by higher-energy collision dissociation (HCD) with the energy set at 25 NCE. Intensity threshold for ions selection was fixed at 1.7 × 10^4^ with charge exclusion of z = 1 and z > 5. The MS/MS spectra were acquired at a resolution of 17,500, with a target value of 2 × 10^5^ ions or a maximum integration time of 120 ms and the isolation window was set at 4.0 *m/z*. The obtained RAW data files were processed with Maxquant version 1.5.3.12 (http://www.biochem.mpg.de/5111795/maxquant) for protein and peptide identification using the Andromeda search engine and the Uniprot proteome for *M. smegmatis* (Proteome ID: UP000000757, 6601 proteins, 14/05/2016). The normal default parameter settings were used for the MS/MS database search, with carbamidomethylation of cysteine residues and acetylation of the protein N-terminus selected as fixed modifications. Trypsin/P was selected as protease, iBAQ quantitation and the ‘match between runs’ feature were enabled. Reverse hits and common contaminants were removed from the identified protein list. Likewise, only protein identifications with a q-value < 0.01 and two or more unique peptides were retained for the further analysis.

### Enrichment analysis

Even though a centrifugation step was used to pellet the cell wall of mycobacteria, this cell wall fraction still contained a high number of cytosolic proteins that are not sufficiently removed during centrifugation. This represents a common problem in cell wall proteomics studies. In order to overcome this challenge, we developed a post-measurement enrichment analysis method to filter out these cytosolic contaminants *in silico*. The key to this approach is the assumption that during cell wall enrichment, the abundance of a true cell wall protein should increase in the cell wall fraction compared to the corresponding cytosolic fraction, whereas the abundance of a true cytosolic protein should decrease in the cell wall fraction compared to the corresponding cytosolic fraction. This can be easily analysed by assessing the abundance of proteins - inferred from their iBAQ values - using the bioinformatic program Perseus. To this end, the Maxquant output file was uploaded in Perseus version 1.5.2.6 (http://www.biochem.mpg.de/5111810/perseus), and reverse hits, common contaminants and hits with q-values >0.01 were filtered out. iBAQ values were then log2-transformed and a standard volcano plot analysis performed with the cytosolic fraction (biological replicates n≥3) as the reference group and the respective cell wall/cell debris fraction as the test group (biological replicates n≥3). We kept the standard values for determining significantly enriched proteins at the default settings of FDR = 0.05 and background variability S_0_ = 0.1. All proteins that exceed this threshold are significantly enriched in the cell wall fraction and, thus, most likely genuine cell wall proteins.

### Method validation

**Fig. 2:**
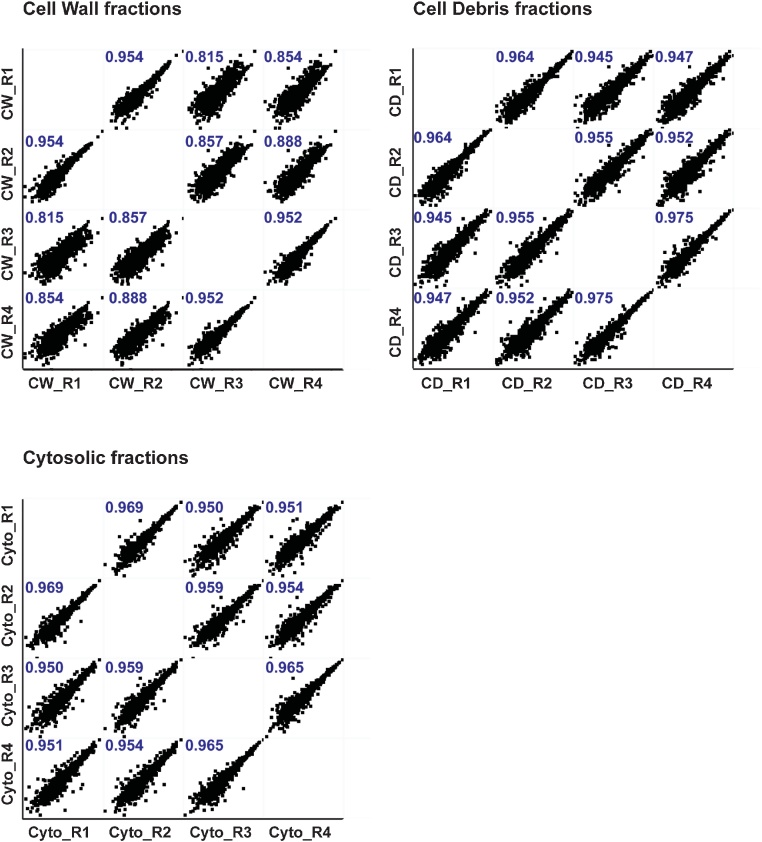
Analysis of reproducibility between group replicates. Scatter plots show log2-transformed iBAQ-values for protein groups identified in all four replicates of one cellular fraction (cell wall fraction, cell debris fraction, cytosolic fraction). The Pearson correlation coefficient is shown for each comparison.

To validate this cell wall sample preparation procedure, we performed the complete protocol with *M. smegmatis* in four biological replicates using the detergent mixture Triton X100, Tween-20 and CHAPS for solubilization of the cell wall fraction. We plotted the log2-transformed iBAQ values of all four biological replicates of each cellular fraction (*i.e*. cytosol, cell wall and cell debris) against each other using a multi scatter plot and calculated the Pearson correlation coefficient for each comparison (Fig. 2). The protein extraction procedures are highly reproducible for all three cellular fractions, with a Pearson correlation coefficient of 0.815–0.954 for the cell wall fraction, 0.945–0.975 for the cell debris fraction and 0.950–0.969 for the cytosolic fraction.

**Fig. 3:**
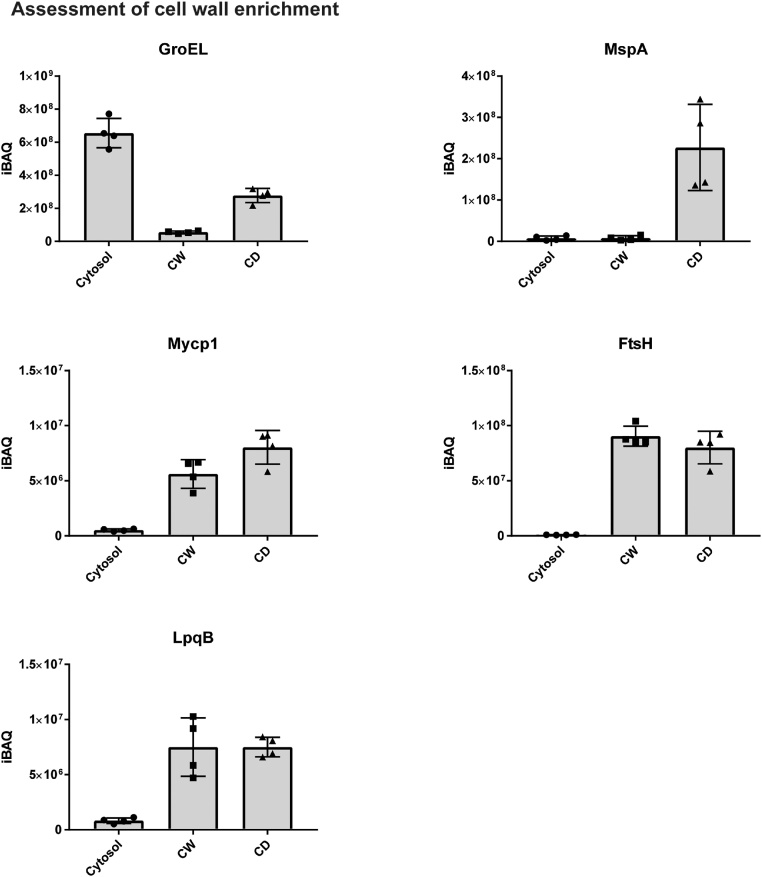
Assessment of cell wall enrichment by known subcellular markers. The abundance of known subcellular markers for the cytosol (GroEL), the outer membrane (MspA), the cell wall/plasma membrane (Mycp1, FstH) and lipoproteins (LpqB) are shown as iBAQ values for the cytosol, the cell wall fraction (CW) and the cell debris fraction (CD).

To further assess whether the cell wall fraction was successfully enriched by differential centrifugation, we made use of the abundances of known subcellular markers for the cytosol or the cell wall. To this end, we assessed the change in abundance between the cytosol and the cell wall or cell debris fraction (judged on the basis of iBAQ values) for GroEL as a cytosolic marker, MspA as a marker for the outer membrane, Mycp1 and FstH as a marker for the cell wall/plasma membrane and LpqB as a marker for the cell wall (Fig. 3). For both the cell wall fraction as well as the cell debris fraction the abundance of GroEL decreased by several orders of magnitude compared to the cytosolic fraction, while the abundance of cell wall markers such as Mycp1, FtsH and LpqB increased dramatically compared to the cytosolic fraction, strongly suggesting that the cell wall proteome was successfully enriched in both the cell wall as well as the cell debris fractions. Interestingly, the outer membrane protein MspA was high in abundance in the cell debris fraction, but nearly absent in the cytosolic and cell wall fraction, which might suggest that the cell debris fraction is able to extract outer membrane proteins more efficiently, but this hypothesis requires further experimental confirmation. Overall, this data confirms that the sample preparation procedures described here are highly reproducible and yield a subset of the total proteome that is highly enriched in cell wall proteins for downstream quantitative analyses.

**Fig. 4:**
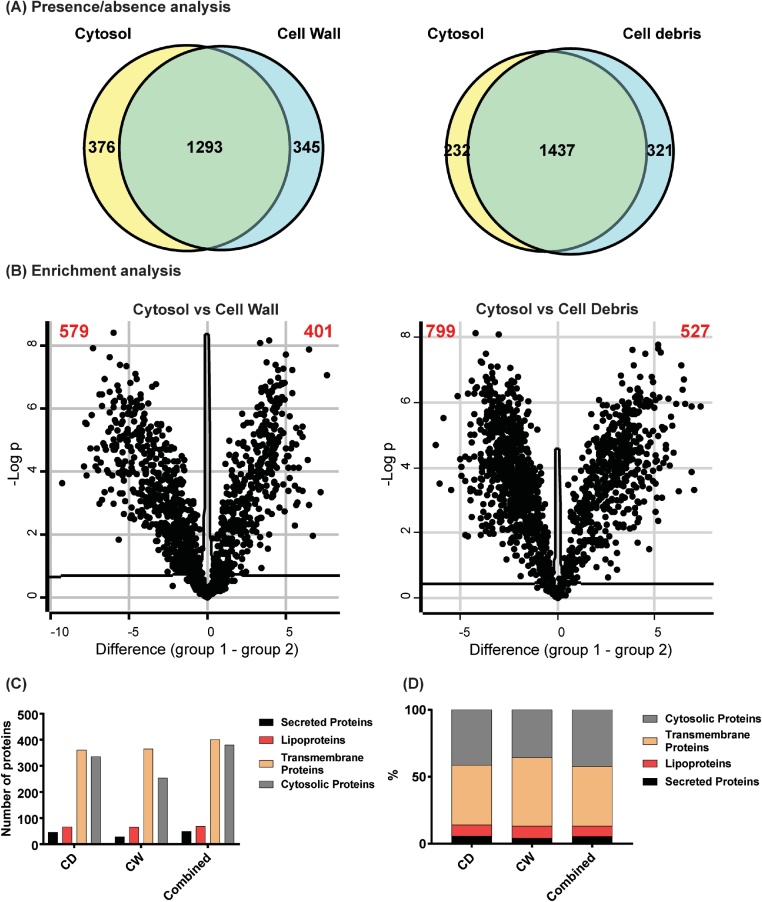
Bioinformatic enrichment analysis identifies subset of genuine cell wall proteins. (A) Presence/absence analysis between the cell wall or cell debris fraction and the cytosol identified 345 (CW) or 321 (CD) proteins that are most likely cell wall proteins. (B) Volcano plot analysis between the cell wall or cell debris fraction and the cytosol identified 401 (CW) and 527 (CD) proteins that are significantly enriched and thus most likely cell wall proteins. iBAQ values were log2-transformed and proteins were defined as significantly enriched if they passed a FDR of 0.05 and an S_0_ of 0.1. (C) The number of proteins that are predicted to be either secreted, a lipoprotein or have a transmembrane helix are shown for the cell wall fraction (CW), the cell debris fraction (CD) and the combination of the two (Combined). (D) The purity of the cell wall fraction, the cell debris fraction and the combination of the two (Combined) is shown as percentage of proteins with a secretion signal, lipobox motif or transmembrane domain within all identified proteins.

As highlighted above, both the cell debris and the cell wall fractions always contain cytosolic proteins that have carried through due to their high abundance in the total cell lysate and to the usually much lower abundance of cell wall proteins. We therefore developed a post-measurement, *in silico* filtering step to identify and remove these carry-over cytosolic proteins from the resultant datasets. As a first step, all proteins identified in the cell wall or cell debris fraction are compared to the cytosol using a presence-absence approach. We defined proteins as present if at least 2 unique peptides were identified per protein with a q-value of <0.01. All proteins that were only present in the cell wall or cell debris fraction – 345 for the cell wall and 321 for the cell debris fraction in this example (Fig. 4A) – are most likely cell wall proteins. Next, all proteins that were identified in both the cell wall or cell debris fraction and the cytosol were used for an enrichment analysis. In this analysis, the log2-transformed iBAQ values of each protein were compared between the cell wall or cell debris and the cytosol using a volcano plot analysis. To define significantly enriched proteins, we used a FDR of 0.05 and a S_0_-value of 0.1 as cut-off. All proteins that are significantly enriched in the cell wall (401) or cell debris fraction (527) compared to the cytosol are most likely cell wall proteins (Fig. 4B). We further combined the list of proteins that are uniquely present in either the cell wall or the cell debris fraction (Fig. 4A) with the list of proteins that are significantly enriched in either the cell wall or the cell debris fraction (Fig. 4B) to yield the final list of cell wall proteins with 746 proteins for the cell wall fraction and 848 proteins for the cell debris fraction. All proteins that were identified in both fractions but which were removed through this *in silico* filtering step are most likely cytosolic carry-over proteins. The purity of the resulting cell wall fractions was further analysed by assessing the number of proteins in each fraction that are predicted to be either secreted proteins (and therefore likely to be transiently present in a cell wall fraction), or to have a lipobox motif (identifying it as a putative lipoprotein), or to have a transmembrane helix.

In total, using the methods described here, we identified 2065 proteins across the three subcellular fractions, of which 899 (∼44%) were found to be significantly enriched in the cell wall or the debris fraction after application of our post-measurement *in silico* filter step. Of these 899 cell wall proteins, 49 were predicted to be secreted, 69 were predicted to be lipoproteins and 401 were predicted to have transmembrane helices (Fig. 4C). However, 380 of these 899 identified proteins (∼42%; Fig. 4D) did not contain any motif that would predict them to be cell wall proteins, yet our post-measurement *in silico* analysis confidently identified them as cell wall proteins, either due to presence/absence or to differential enrichment considerations (*vide supra*); this serves to highlight the well-known limitations of current motif-based theoretical cellular localisation prediction tools and demonstrates the value of the robust, reproducible experimental method to identify and quantify cell wall proteins in mycobacteria that are described here.

## Supplementary material and/or Additional information

The dataset used in this publication is part of a larger dataset freely accessibly on PRIDE (Accession Number: PXD008075).
